# Awareness, adoption readiness and barriers to the uptake of downstream space technologies among European SMEs: Findings from a cross-sectoral survey

**DOI:** 10.12688/openreseurope.24431.1

**Published:** 2026-07-25

**Authors:** Michail Mitsouridis, Paraskevi Rampota, Christina Balla

**Affiliations:** 1 International Projects and Studies, Q-PLAN INTERNATIONAL ADVISORS PC, Thessaloniki, 55133, Greece

**Keywords:** Downstream Space Technologies, Circular Economy, Space Data, Small and medium enterprises (SMEs), Start-ups, Scale-ups, Industry, Green Transition

## Abstract

Downstream space technologies, including Earth Observation, Global Navigation Satellite Systems (GNSS), and Satellite Communications, have considerable potential to support environmental monitoring, resource efficiency, and the green transition of European industry. However, evidence on the awareness, readiness, and adoption of these technologies among European small and medium-sized enterprises (SMEs) remains limited. To address this gap, a cross-sectional survey was conducted on 503 respondents representing organisations across the 14 European industrial ecosystems. The survey examined sustainability practices, awareness and use of downstream space technologies, organisational readiness, perceived relevance, and barriers to adoption. The results show that sustainability is becoming an important strategic priority, with many organisations already implementing sustainability-focused solutions or planning to do so. In contrast, awareness and uptake of downstream space technologies remain low. Only a small proportion of respondents actively use space-based data and services, while many organisations report limited familiarity, insufficient technical expertise, and uncertainty regarding their business value. The most frequently identified barriers are unclear return on investment, implementation costs, lack of awareness of available solutions, and limited internal capabilities. Differences are also observed across company types and industrial ecosystems, with medium-sized enterprises generally demonstrating higher levels of awareness and readiness than smaller firms. These findings provide cross-sectoral assessments of awareness, adoption readiness, and barriers to the uptake of downstream space technologies among European SMEs, offering an evidence base to inform future research and innovation support activities.

## Introduction

The continuous evolution of downstream space technologies is unlocking significant opportunities for innovative applications that reinforce European industrial leadership and accelerate the transition towards climate neutrality. In the context of the circular economy, Earth Observation and GNSS have demonstrated their capacity to optimise resource use, enhance the resilience and efficiency of value chains, and support more effective environmental monitoring. Despite this considerable potential, according to a 2023 survey conducted by EUSPA the uptake of space-based data remains low, with only approximately 7% of European SMEs integrating such information into their environmental operations. ^1^

To assess the preparedness of SMEs to leverage downstream space technologies for the green transition, a comprehensive survey was administered across multiple European countries. The survey evaluated five interrelated dimensions: awareness of the potential of downstream space technologies; organisational readiness, including technical capabilities and workforce skills for integrating space-derived data; the current implementation of sustainability-focused practices; the integration of environmental monitoring into business operations; and the barriers affecting adoption of space data and services. Together, these dimensions provide a multidimensional assessment of SMEs’ capacity to exploit downstream space technologies as enablers of sustainable innovation and competitiveness.

## Methodology and research limitations

This survey gathered insights from 503 individuals working in entities across all 14 EU industrial ecosystems, namely Aerospace & Defense, Agri-food, Construction, Creative & Cultural, Digital, Electronics, Energy-intensive, Health, Mobility, Transport & Automotive, Proximity, Social Economy & Civil Security, Renewable Energy, Textiles, and Tourism. ^2^ To ensure holistic thematic coverage, the questionnaire was structured into the following sections:

• Sustainability Practices & Environmental Impact
• Awareness & Adoption of Downstream Space Data
• Barriers & Support Needs

Prolific ^3^ was selected as the primary data collection platform for the survey, chosen for its extensive reach and ability to gather responses from SMEs through integrated screening. Written informed consent was obtained electronically from all survey participants prior to their participation in the survey and before the analysis of their responses.

The respondent sample comprises individuals from various European countries, with notable participation from Poland (13.4%), Germany (12.5%), Spain (11.7%), Italy (11.6%), France (8.6%), Greece (7.0%), and the Netherlands (5.9%), along with other countries, each contributing less than 5%. While the survey shows higher participation from certain countries, it is important to note that these rates do not necessarily reflect a more developed space ecosystem in them. Factors such as population size and the selected survey distribution method likely influenced the participation rates. In terms of employment type, 51.0% of respondents worked in medium enterprises (50–249 employees), 35.8% in small enterprises (<50 employees), 6.8% in scale-ups, and 6.4% in start-ups.

Moreover, survey responses are primarily concentrated in the Digital & IT Services sector (33.4%), followed by the Cultural & Creative (9.5%). Other sectors with notable participation include Health (6.8%), Tourism (7.0%), Energy-intensive (6.4%), Mobility, Transport & Automotive (6.8%), Electronics & Telecommunications (5.6%), Construction (6.4%), and Agri-food (6.8%). The “Other” category (7.0%) mainly includes respondents from the mechanical and machine, financial services, and the education sector. Despite the broad sectoral representation in the survey, certain limitations should be acknowledged. Notably, some key industrial domains are underrepresented. For example, the Aerospace and Defence sector accounts for only 8 out of 503 total respondents, which limits the generalizability of findings to stakeholders in this domain. This uneven distribution may introduce bias in interpreting sector-specific needs, challenges, and opportunities, and should be considered when extrapolating insights across the broader industrial landscape.

In all figures below, the survey results follow the same presentation pattern, using 5 pie charts to represent: the overall dataset (all enterprises), Scale-ups, Start-ups, Small enterprises, and Medium enterprises.

## Results of the analysis

### Sustainability practices & environmental impact

**Figure 1.**
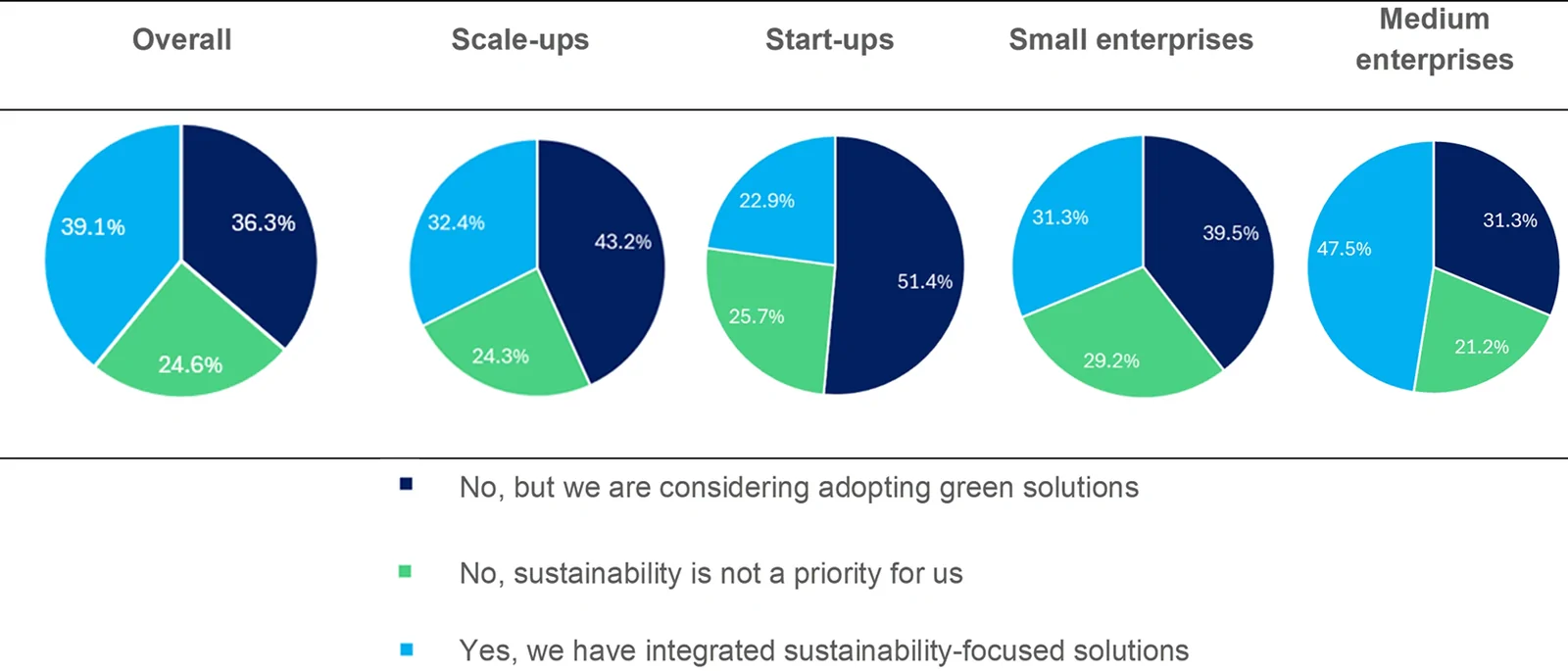
Current Use of sustainable technologies.

Sustainability is becoming a strategic imperative for companies across sectors, driven by evolving EU regulations such as the Corporate Sustainability Reporting Directive, ^4^ increasing consumer demand, and shifting investor expectations. Evaluating the extent to which companies integrate sustainable technologies enables a better understanding of actual practices, existing limitations, and areas where targeted support may be needed to facilitate broader adoption. This section explores how companies position themselves within this green transition. Survey participants were initially asked whether they use sustainable solutions. Figure 1 shows a comprehensive overview of the adoption of sustainable technologies per employment type.

According to the “Overall” pie chart, 39.1% of the enterprises have already integrated sustainability-focused solutions, indicating a proactive approach towards environmental responsibility. Meanwhile, 36.3% are considering adopting green solutions, reflecting a growing awareness and willingness to embrace sustainability practices. However, 24.6% of respondents do not prioritize sustainability.

Breaking down the results by company type reveals several notable differences. Start-ups demonstrate the highest openness to adopting green practices, with 51.4% considering adoption, but only 22.9% having implemented them to date, indicating strong awareness but limited internal capacity. Scale-ups appear more advanced, with 32.4% already using sustainable solutions and 43.2% actively considering them. Small enterprises are relatively balanced, as 31.3% have implemented such practices and 39.5% are planning to, whilst medium enterprises lead in actual implementation, with 47.5% reporting adoption, though 21.2% still do not view sustainability as a priority.

When analysed by EU industrial ecosystem, 50% or more of the Agri-food, Electronics & Telecommunications, Energy-intensive, Mobility - Transport and Automotive, Proximity - Social Economy & Civil Security, and Textile sectors have already implemented sustainability-focused solutions.

**Figure 2.**
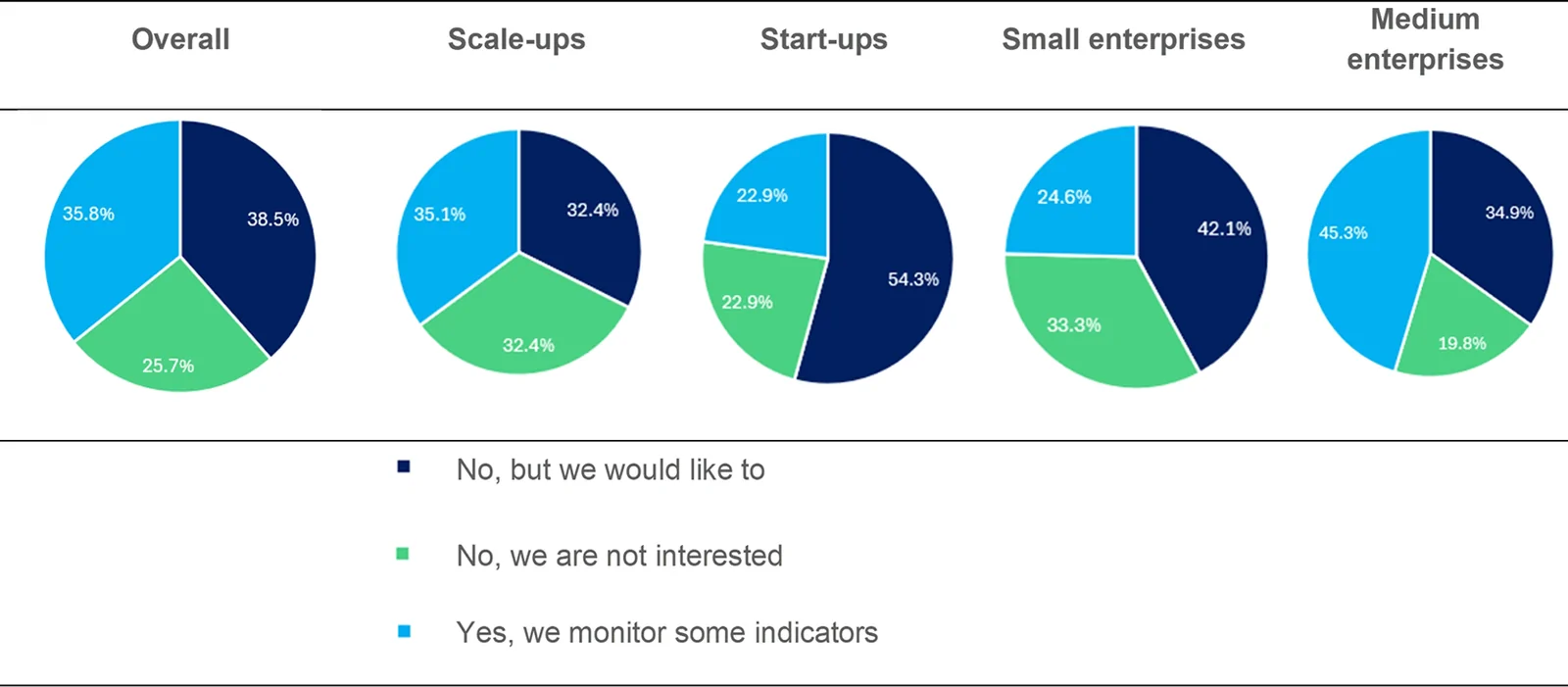
Engagement in environmental monitoring.

Figure 2 further complements these findings by showcasing the levels of engagement in monitoring environmental indicators per employment type.

Overall, a significant portion of companies (35.8%) already tracks relevant indicators, suggesting a degree of operational readiness or existing internal processes. Interestingly, a slightly larger share (38.5%) expressed interest in environmental monitoring but have not yet implemented it, which may reflect uncertainty around technical feasibility, cost, or resource availability. Meanwhile, 25.7% of respondents indicated no engagement in such practices. By company type, start-ups show strong intent to begin environmental monitoring, with over half (54.3%) expressing interest, though only 22.9% currently track indicators. Medium enterprises are the most engaged, with 45.3% already monitoring environmental indicators.

When analysed by EU industrial ecosystem, 50% or more of the Agri-food, Electronics & Telecommunications, and Proximity - Social Economy and Civil Security sectors are currently monitoring some environmental indicators.

These findings highlight awareness and willingness among European enterprises to integrate sustainability-focused solutions and environmental monitoring into their operations. However, the pace of adoption varies by company type, with start-ups showing strong interest but facing barriers to implementation, while medium enterprises lead in actual adoption. When analysed by sector, Agri-food, Electronics & Telecommunications, Energy-intensive, Mobility - Transport and Automotive, Proximity - Social Economy & Civil Security, and Textile have already implemented sustainability solutions, while Culture & Creative and Renewable Energy companies are considering them. To accelerate broader uptake, targeted support strategies, such as financial incentives, clearer regulatory frameworks, and accessible technical resources, are essential. Additionally, enterprises that do not currently prioritize sustainability may benefit from stronger communication on its strategic value to act.

### Awareness & adoption of downstream space data

**Figure 3.**
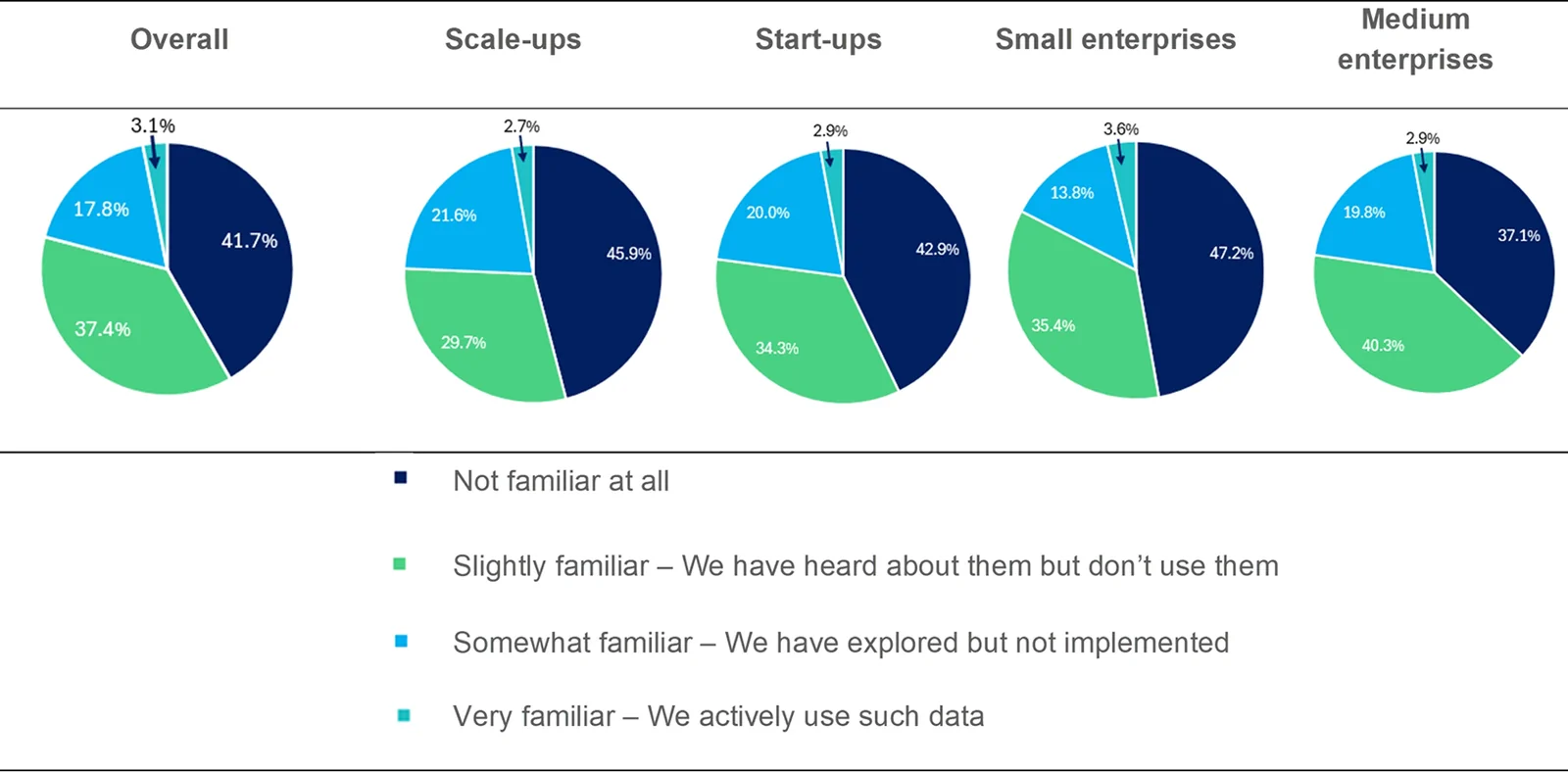
Awareness of space data and services.

While sustainability is gaining ground downstream space technologies present a promising and still expanding opportunity to accelerate progress toward the Sustainable Development Goals. ^5^ This section introduces survey findings on companies’ familiarity with space-based data and services, their capacity to adopt them, and their interest in technologies such as Earth Observation, Satellite Communication, and GNSS. Figure 3 illustrates the awareness levels of companies regarding space data and services, categorizing respondents based on their familiarity with space data and services.

Overall, 17.8% of the respondents have explored the potential uses of downstream space data and services but have not yet implemented any of those. A significant portion (37.4%) has heard about downstream space data and services but does not currently use them. The largest group (41.7%) is not familiar with downstream space data and services at all, whilst only a small segment (3.1%) indicates that they are very familiar with downstream space data and services and actively use them in their operations. Looking at different company types, medium enterprises appear more advanced in terms of awareness: over 40% are slightly familiar with space data and services, and nearly 20% report some level of exploration. Small enterprises have the highest shares of respondents with no familiarity at all (>47%), while actual usage across all categories remains very low, at around 2–3%.

When broken down by EU industrial ecosystem, at least 39% of companies in the Agri-food, Construction, Cultural & Creative, Digital & IT Services, Health, Retail, and Tourism sectors are entirely unfamiliar with downstream space data. Even in more technology-driven sectors such as Aerospace & Defense and Mobility, Transport & Automotive, active usage remains limited to 11.1% and 14.7%, respectively, with no other sector surpassing 8%.

**Figure 4.**
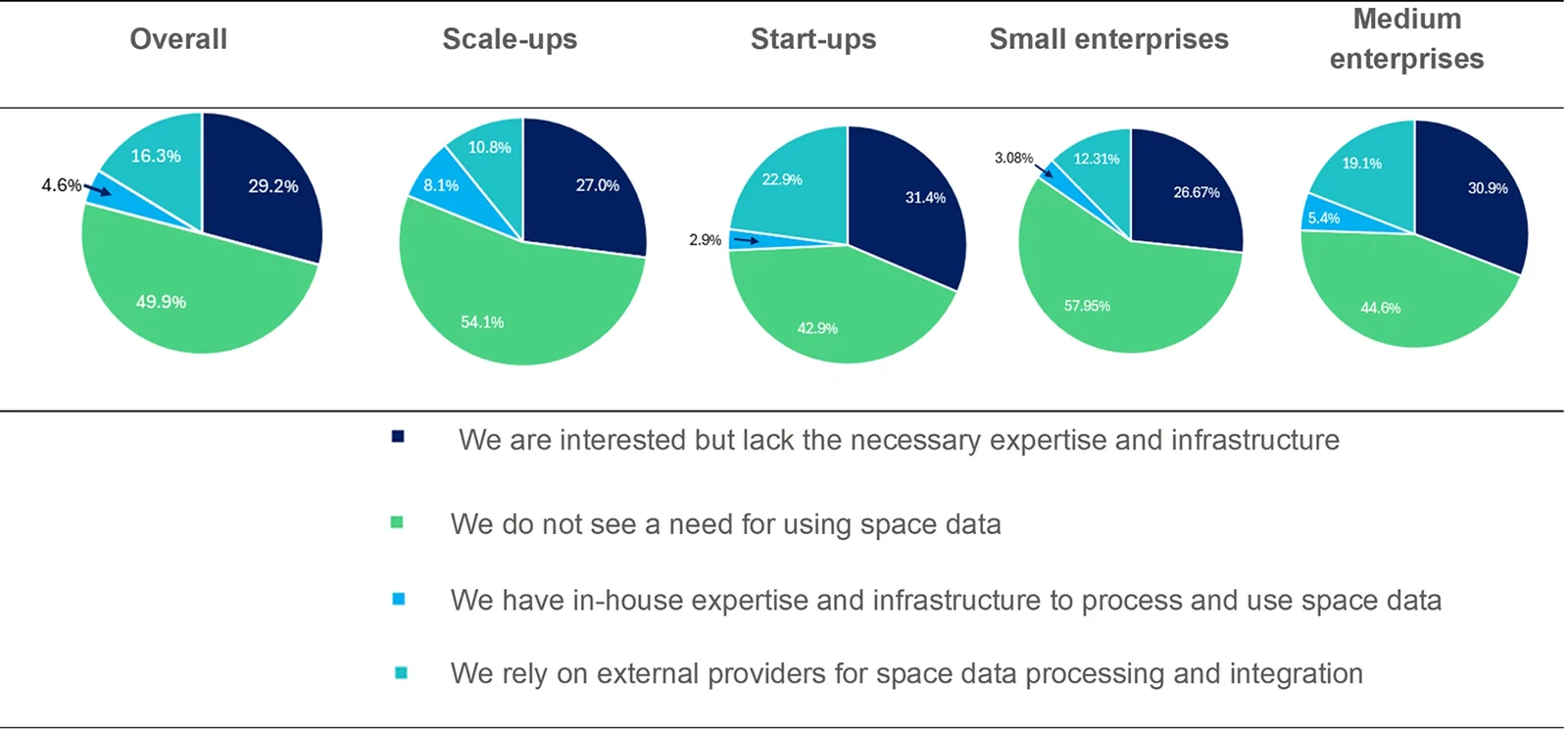
Readiness to use space data.

Building on these insights, figure 4 delves into the expertise and infrastructure available for processing and using space data. This figure provides a clearer picture of how equipped different entities are to handle space data, which is essential for effective utilization.

The overall results indicate that a small portion of respondents (4.6%) are well-equipped with in-house expertise and infrastructure, while 16.3% depends on external providers. A significant portion (29.2%) show interest but face challenges due to a lack of expertise and infrastructure and surprisingly, half of the respondents do not perceive a need for using space data. By company type, scale-ups and medium enterprises appear to be more advanced, with 8.1% and 5.4% respectively reporting in-house expertise. Moreover, 19.1% of medium enterprises use external providers, the highest percentage among all company types. By EU industrial ecosystem, over 75% of companies in the Construction, Cultural & Creative Digital & IT Services, Energy-intensive, Health, Retail, and Tourism sectors have neither in-house expertise nor rely on external providers for space data processing and integration.

**Figure 5.**
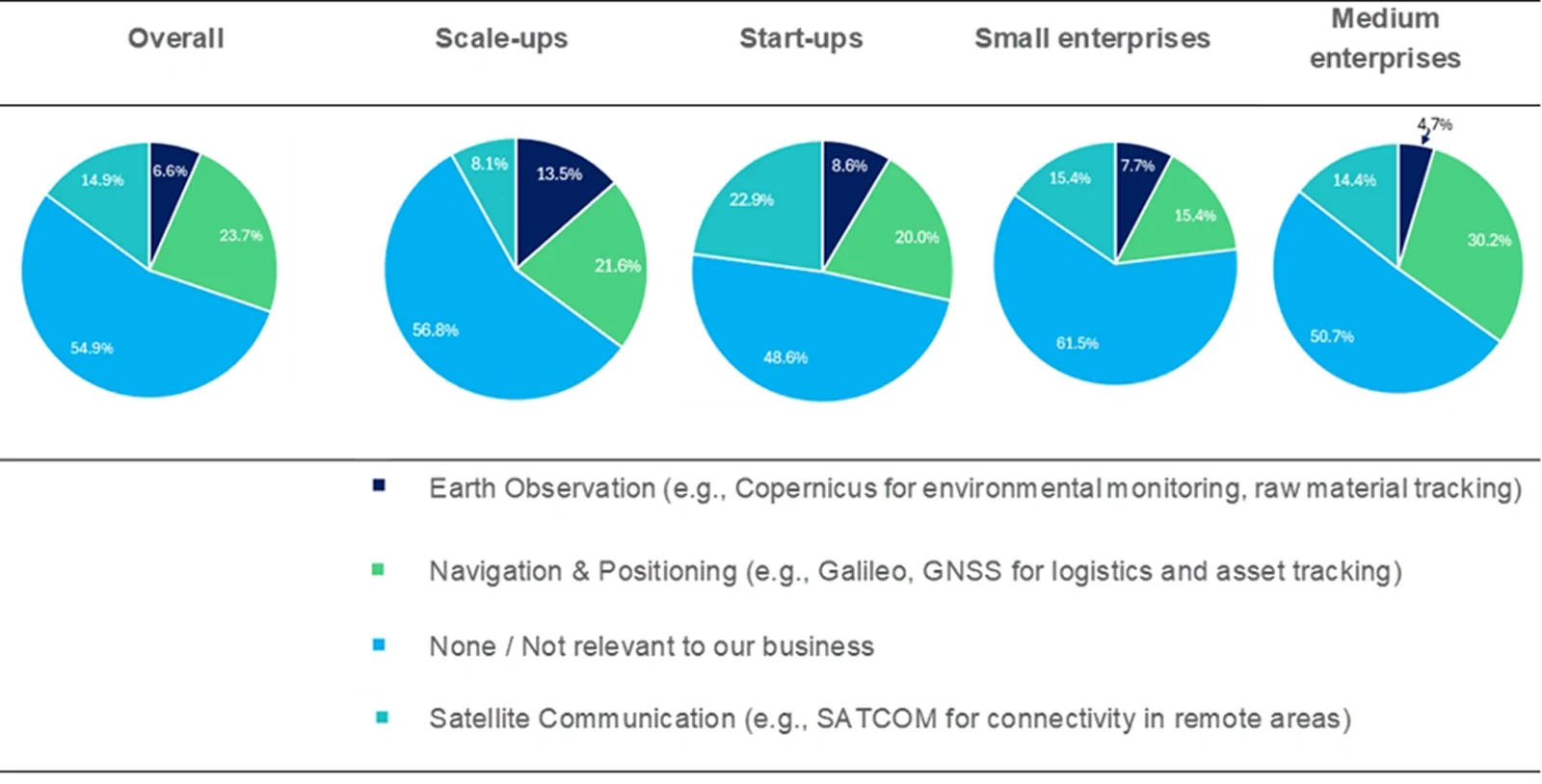
Interest in space technologies.

Building on the discussion of expertise and infrastructure, the focus now shifts to businesses’ interest in specific space technologies. Figure 5 illustrates how different types of businesses perceive its relevance and potential for their operations.

Overall, only 6.6% of respondents show interest in Earth Observation technologies. Satellite Communication used for remote connectivity, attracts 14.9%, while 23.7% focus on Navigation & Positioning. Nonetheless, the majority (54.9%) consider space technologies irrelevant to their business. Scale-ups exhibit a higher interest in Earth Observation (13.5%), start-ups show notable interest in Satellite Communication (22.9%), and medium enterprises show the highest interest (30.2%) in Navigation & Positioning. Across EU industrial ecosystems, over 30% of companies in the Agri-food, Construction, Mobility – Transport & Automotive, Proximity – Social Economy & Civil Security, Renewable Energy, and Retail sectors show interest in Navigation & Positioning. Interest in other space technologies is lower, except for Satellite Communication, which is reasonably relevant for Electronics & Telecommunications companies.

**Figure 6.**
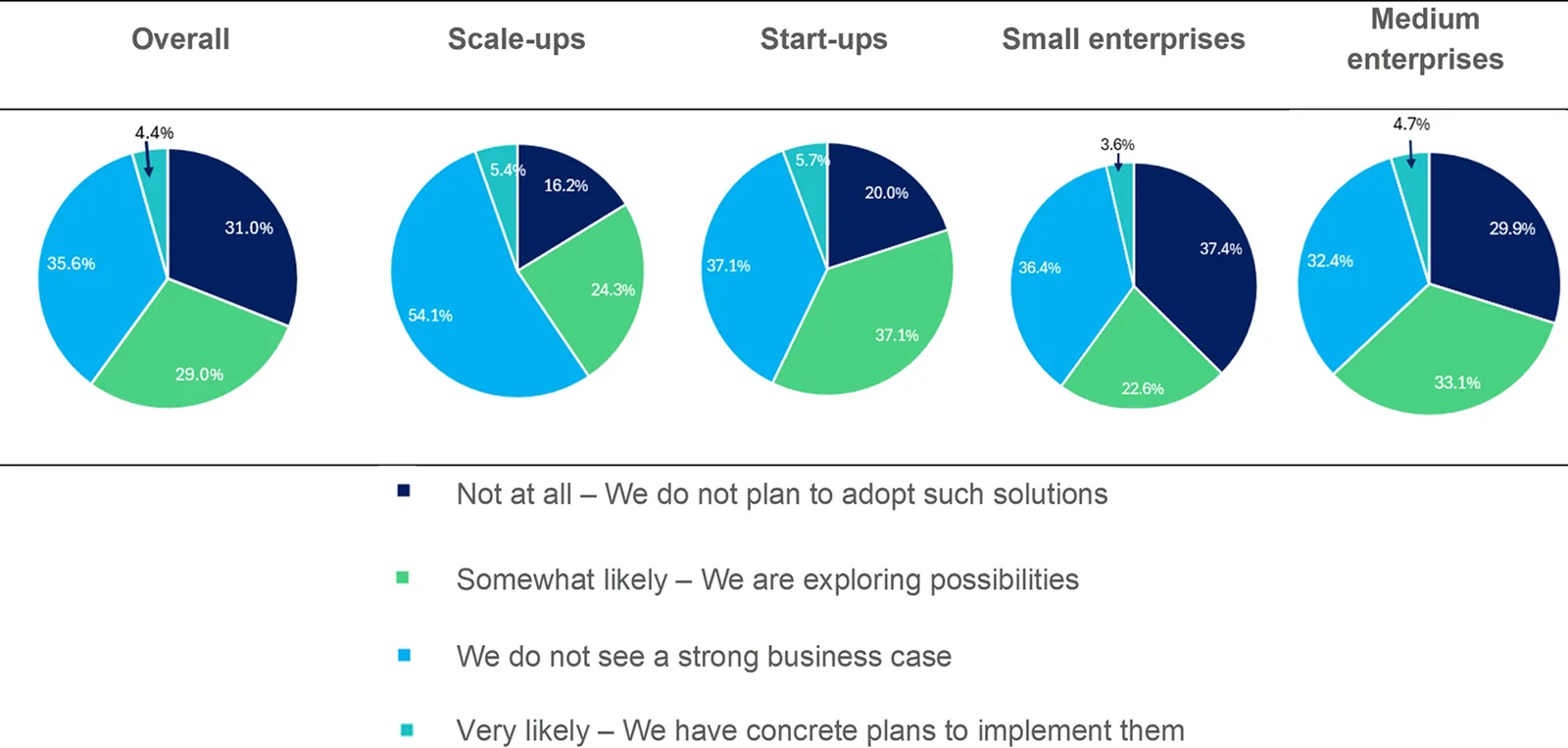
Future outlook on space-enabled sustainability.

Examining next the future outlook on space-enabled sustainability solutions among businesses, survey participants were asked to examine the likelihood of implementing these solutions, offering a glimpse into the potential adoption trends. The summary of their responses is shown in Figure 6.

Overall, the results indicate that only a small portion (4.4%) of respondents have concrete plans to implement space-enabled sustainability solutions, while 29.0% is exploring possibilities. A significant number (35.6%) do not see a strong business case for these solutions, and 31.0% do not plan to adopt them at all. Across different company types, scale-ups appear the most sceptical, with 54.1% indicating they do not see a strong business case for adopting space-enabled sustainability solutions. Small enterprises remain the most hesitant, with 37.4% stating they do not plan to adopt such solutions and just 3.6% indicate readiness to implement them. Adoption potential is more pronounced for some industrial sectors. Indicatively, more than half of the companies in Aerospace & Defense, Electronics & Telecommunications, Proximity – Social Economy & Civil Security, and Renewable Energy are either actively exploring opportunities or have concrete implementation plans, indicating stronger sector-specific drivers for space-enabled sustainability solutions.

The findings reveal a generally low level of familiarity with and adoption of downstream space data and services among European enterprises. While some companies, particularly medium enterprises and scale-ups, demonstrate higher awareness and limited in-house expertise, the vast majority remain unfamiliar with space technologies or perceive them as irrelevant to their business. Interest in specific applications, such as Navigation & Positioning and Satellite Communication, is more pronounced among certain company types, yet actual implementation remains rare. Additionally, the adoption of space-enabled sustainability solutions faces significant barriers, with many companies struggling to identify a strong business case. At the sectoral level, awareness and expertise gaps are particularly evident in Agri-food, Construction, Cultural & Creative, Digital & IT Services, Health, Renewable Energy, Retail, and Tourism, where companies are entirely unfamiliar with space data, and the majority lacks both in-house capabilities and external support. In Aerospace & Defense, Electronics & Telecommunications, Proximity – Social Economy & Civil Security, and Renewable Energy on the contrary, most of the companies are already exploring or planning implementation. To bridge the existing gaps, targeted awareness campaigns, sector-specific technical support, and clear demonstrations of business value showcasing tangible benefits and potential returns on investment will be essential in driving wider adoption across companies.

### Barriers and support needs

Despite the growing potential of space-enabled solutions, many companies encounter significant challenges to adoption. ^6^ Understanding these challenges, as well as the type of support companies are looking for, can help fine-tune future innovation support programs. This section identifies key barriers and highlights support needs.

**Figure 7.**
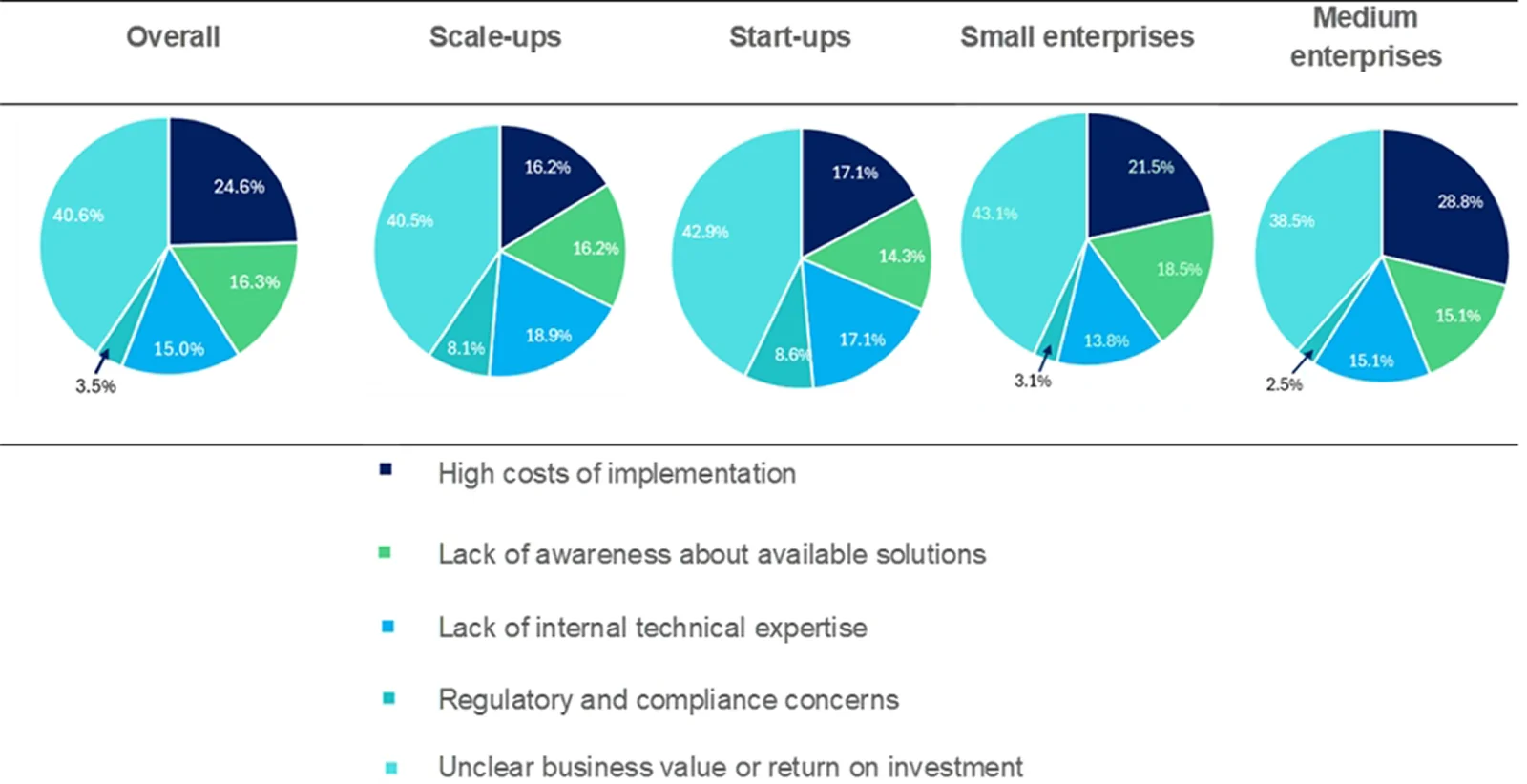
The biggest barriers preventing adoption of space data.

Building on the findings about adoption intentions, the following graph provides insight into what currently holds companies back. Figure 7 presents respondents’ selection of 5 potential barriers that may hinder adoption of space data across different types of enterprises.

In total, the results reveal that regulatory and compliance concerns, although less prominent (3.5%), still represent a barrier for some businesses. Lack of internal technical expertise (15.0%) and lack of awareness about available solutions (16.3%) are notable barriers. High costs of implementation discourage 24.6% of respondents, suggesting that the initial investment required for space data solutions is a significant deterrent, while the most significant challenge is the unclear business value or return on investment, cited by 40.6% of respondents. This also poses a strategic risk for technology providers and public stakeholders, as uncertainty around perceived value may lead to stagnation in demand. Interestingly, regulatory and compliance concerns were considerably higher among scale-ups and start-ups, while high costs of implementation were reported more frequently by small and medium enterprises.

Across various EU industrial ecosystems, over 30% of companies in Electronics & Telecommunications, Mobility – Transport & Automotive, Proximity – Social Economy & Civil Security, Renewable Energy, and Tourism are deterred by high implementation costs. Additionally, more than 20% of companies in the Construction, Energy-intensive, Health, and Mobility – Transport & Automotive sectors lack awareness of available solutions. Similarly, over 20% of companies in Agri-food, Electronics & Telecommunications, Renewable Energy, and Tourism face challenges due to a lack of internal technical expertise. Furthermore, more than 40% of companies in Aerospace & Defence, Agri-food, Digital & IT services, Health, Retail, and Textiles cite unclear business value or return on investment as significant barriers. This trend poses a latent risk of uneven adoption across sectors, potentially amplifying digital divides and slowing ecosystem-wide innovation.

**Figure 8.**
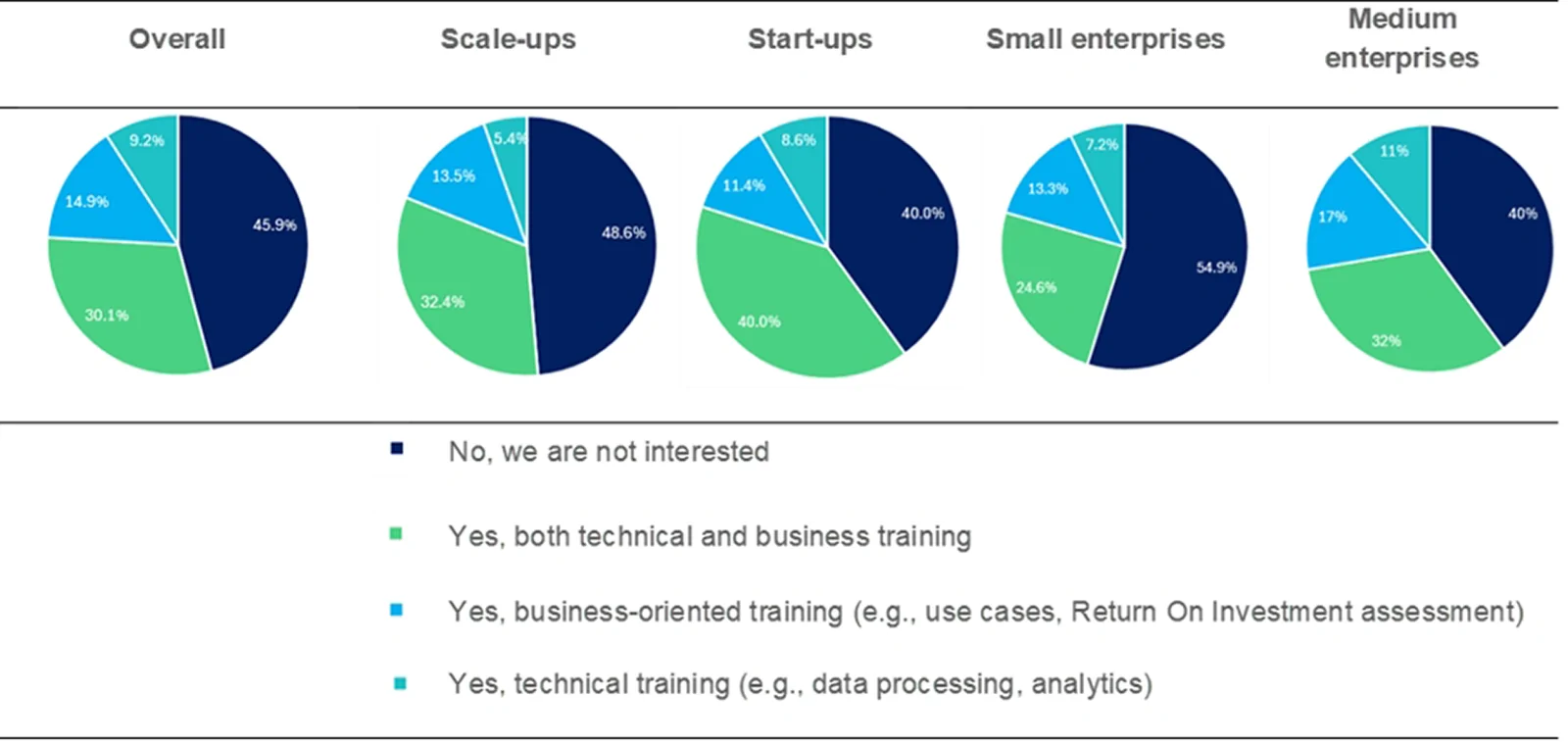
Interest in support for space data integration.

Beyond identifying barriers to adoption, it is also important to understand the type of support that companies consider useful for building internal capacity. Figure 8 below illustrates the extent to which companies are open to technical- and business-oriented training.

The overall results show varied perspectives on training for space data integration. While 45.9% of respondents reported no current interest in training, the remaining share expressed demand for a combination of technical and business-focused support (30.1%). Smaller groups indicated interest in either business-oriented training (14.9%) or technical skills development (9.2%), pointing to more specific needs. A closer look across company types reveals that start-ups and medium enterprises are the most receptive to training, whilst small enterprises are the least engaged, with 54.9% expressing no interest. The high level of disinterest in training, particularly among small enterprises, may reflect a risk of skills stagnation, with long-term implications for innovation capacity.

Interest in space data integration training also varies significantly across EU industrial ecosystems. Over 20% of companies in the Energy-intensive and Renewable Energy sectors are interested in technical training. More than 20% of businesses in Aerospace & Defense, Agri-food, Electronics & Telecommunications, Mobility – Transport & Automotive, Proximity – Social Economy & Civil Security, Textiles, and Tourism are seeking business-focused training. Additionally, over 40% of companies in Electronics & Telecommunications and Retail sectors are interested in both technical and business training.

The findings highlight key barriers to the adoption of space data solutions, with unclear business value emerging as the most significant challenge, followed by high implementation costs and limited technical expertise. While regulatory concerns are less prominent overall, they are more pronounced among start-ups and scale-ups. When broken down by industrial ecosystem, high implementation costs deter over 30% of companies in Electronics & Telecommunications, Mobility – Transport & Automotive, Proximity – Social Economy & Civil Security, Renewable Energy, and Tourism. More than 20% of companies in Construction, Energy-intensive, Health, and Mobility lack awareness of available solutions, and over 20% in Agri-food, Electronics & Telecommunications, Renewable Energy, and Tourism face challenges due to a lack of internal technical expertise. If left unaddressed, these barriers pose cumulative risks that could limit the reach and impact of public and private investments in the space economy. The demand for training is mixed, with nearly half of respondents uninterested, while the rest express a need for both technical and business-oriented support. Notably, start-ups and medium enterprises show the highest willingness to engage in training, while small enterprises remain the least receptive. Addressing these challenges through targeted awareness campaigns, financial incentives, and blended training programs will be essential to fostering broader adoption and maximizing the potential of space data solutions.

## Conclusions

Overall, this survey study contributes to a better understanding of the current landscape regarding awareness, readiness, and adoption of downstream space technologies across European industrial ecosystems. The results demonstrate both the opportunities and the challenges associated with the wider integration of space-enabled solutions in support of sustainability objectives. The evidence generated through this survey can inform future initiatives aimed at strengthening the connection between the space sector and industrial end users. By supporting a closer alignment between technological capabilities and business needs, such efforts can contribute to the broader adoption of downstream space technologies and enhance their role in advancing Europe’s green and digital transitions.

## Ethics and consent

Ethical approval was not required for this study. This research followed the provisions described in the **FIERCE Data Management Plan (Deliverable D7.2 – Data Management Plan – 1st period, Initial version)** to ensure the fair and sound management of research data. The Data Management Plan has been reviewed and accepted by the European Commission and is publicly available at: https://project-fierce.eu/wp-content/uploads/2025/02/D7.2_FIERCE_DMP_v1.pdf

Informed consent procedures were implemented, and written consent was obtained for all survey participants prior to participation. No minors were surveyed.

## Data Availability

The dataset underpinning the analysis is uploaded on Zenodo with DOI: https://doi.org/10.5281/zenodo.15516626. ^7^ Available under the license Creative Commons Attribution 4.0 International.
