# Supplementary material for: Awareness, adoption readiness and barriers to the uptake of downstream space technologies among European SMEs: Findings from a cross-sectoral survey
Source: Open Res Eur. 2026 Jul 25;6:260. [Version 1] doi: 10.12688/openreseurope.24431.1 (PMC13562967; doi:10.12688/openreseurope.24431.1)
Supplement: Peer review history

# Reviewer report: Reviewer response for version 1

**DOI:** 10.21956/openreseurope.26457.r78137

**Published:** 2026-09-07

**Authors:** Francisco Villegas Pinuer

**Affiliations:** 1 Universidad Diego Portales, Santiago, Chile

The introduction should be strengthened to reflect companies' current adoption of the circular economy. For example, the steps SMEs take to adopt these technologies differ: SMEs adopt low-circularity practices, such as recycling, rather than high-circularity practices, such as reducing. In this regard, the section should remark on the relevance of integrating these technologies into SMEs' operations. The approach should consider the potential to develop competitive advantages.

 The methodology should include the steps of the process analysis.

 The results section should compare company sizes. Note differences in the sample's structural conditions.  Given the data, bivariate analysis can enrich the presentation by examining the potential relationship between the established variables.

 The publication would benefit from a stronger discussion section, including specific practical implications for SMEs.

 The conclusions should include a substantive discussion of the identified bridges. In this regard, it should also discuss the process to facilitate their adoption.

Is the study design appropriate and does the work have academic merit?

Partly

Is the work clearly and accurately presented and does it cite the current literature?

Yes

If applicable, is the statistical analysis and its interpretation appropriate?

Yes

Are all the source data underlying the results available to ensure full reproducibility?

Yes

Are the conclusions drawn adequately supported by the results?

Partly

Are sufficient details of methods and analysis provided to allow replication by others?

Partly

Reviewer Expertise:

Environmental sustainability and circular economy

I confirm that I have read this submission and believe that I have an appropriate level of expertise to confirm that it is of an acceptable scientific standard, however I have significant reservations, as outlined above.

# Reviewer report: Reviewer response for version 1

**DOI:** 10.21956/openreseurope.26457.r78132

**Published:** 2026-08-12

**Authors:** Grigorios L. Kyriakopoulos

**Affiliations:** 1 National Technical University of Athens, Athens, Greece

1. The results of the study are presented in Abstract in a rather quantitative way, while a more specific numerical-based specification should be added to better justify them: “ *The results show that sustainability is becoming an important strategic priority, with many organisations already implementing sustainability-focused solutions or planning to do so. In contrast, awareness and uptake of downstream space technologies remain low. Only a small proportion of respondents actively use space-based data and services, while many organisations report limited familiarity, insufficient technical expertise, and uncertainty regarding their business value* ”. These statements can be better communicated with the readers of the study in a more quantitative manner. Other numerical data that are missing from the Abstract section refer to the “ *This survey gathered insights from 503 individuals* ”: calendar year of the survey took place? response rate of the initial respondents’ invited? brief descriptive statistics of the sample: age-sex profile, education profile, contribution per each one of the 14 European industrial ecosystems participated?
2. Based on the following statements of the 1.Introduction section “ *innovative applications that reinforce European industrial leadership and accelerate the transition towards climate neutrality* ”, “ *To assess the preparedness of SMEs to leverage downstream space technologies for the green transition* ”, there should be a more solidified theoretical background on the a) chronological evolution among EU countries, b) geographical-territorial clustering of similar macro-economic characteristics among EU countries, focusing on the transformation of European SMEs space technologies and green economies/priorities/guidance/regulations. This theoretical enhancement could be both a) normative, institutional, regulatory, and/or b) technological-innovation-based. The inclusion of 8-10 more and recently published citations, in enhancing the existing length of section 1. Introduction, or by developing another missing section 2. Literature Review are recommended.
3. All numerical part of the study (absolute numbers, percentages), included in section “ *Methodology and research limitations* ”, has to be presented in the form of Tables for comparison reason, not in plain text. The table-formatted data can be better monitored and traceable from the readers of the study, something that is perplexing when reading them ordered only in plain text.
4. The current situation and a better understanding of why it is important to adopt and to maximize the space data solutions could be more comprehensive in alignment with the development of a new separate main section “Discussion”. In this new section there should be a narrative expansion of existing section “ *Barriers and support needs* ” in which the numerical findings of Figures-pie-diagrams cannot be purely developed per type-size of companies, but also in terms of a) geographical clustering of outcomes among the 14 specific EU countries examined, b) time-span, inferring to the developmental or impeding characteristics reported through time regarding the examined “ *downstream space technologies among European SMEs* ”. This new *Discussion* section can be extended up to one text page, having also cross-citing studies included.

Is the study design appropriate and does the work have academic merit?

Yes

Is the work clearly and accurately presented and does it cite the current literature?

Yes

If applicable, is the statistical analysis and its interpretation appropriate?

Yes

Are all the source data underlying the results available to ensure full reproducibility?

Yes

Are the conclusions drawn adequately supported by the results?

Partly

Are sufficient details of methods and analysis provided to allow replication by others?

Yes

Reviewer Expertise:

engineering, environment, renewable energy sources and technologies, business and management, circular economy, low carbon economy, remediation of polluted water sources, analytical techniques

I confirm that I have read this submission and believe that I have an appropriate level of expertise to confirm that it is of an acceptable scientific standard.

# Reviewer report: Reviewer response for version 1

**DOI:** 10.21956/openreseurope.26457.r78134

**Published:** 2026-08-07

**Authors:** Maria Bartekova

**Affiliations:** 1 Bratsilava University of Economics and Business, Bratislava, Slovakia

1. The literature review is insufficient and needs to be significantly expanded

 This is the most significant shortcoming of the manuscript. The introduction is very brief and relies mainly on institutional reports and policy documents. The bibliography contains only seven sources, most of which are materials from the European Commission, EU agencies, the UN or project materials. Academic peer-reviewed literature is minimally represented.

 The manuscript should include a separate and comprehensive literature review, focusing in particular on the following areas:

 Adoption of downstream space technologies and space-based services;

- Use of Earth observation, GNSS and satellite communications in SMEs;
- Adoption of digital and green technologies by SMEs;
- Organizational readiness and technological capabilities;
- Perceived utility, business value and return on investment;
- Barriers to adoption of innovations, including costs, skills shortages, regulation and uncertainty;
- differences between startups, scaleups, small and medium-sized enterprises;
- the contribution of space technologies to sustainability and the circular economy.

Authors should also develop an explicit theoretical or conceptual framework. Examples of suitable frameworks include the Technology–Organization–Environment framework, the technology adoption model, the theory of diffusion of innovation, the concept of absorptive capacity, or organizational readiness for change. The chosen framework should guide the formulation of research questions, the selection of variables, and the interpretation of results.

 A separate discussion must compare the results with previous empirical studies. Currently, the article mostly repeats the percentage results of the survey and formulates recommendations without demonstrating whether the results confirm, challenge, or extend the existing literature.

 2. Research gap, objectives and research questions are not well defined

 The manuscript states that the evidence regarding awareness and adoption of these technologies among European SMEs is limited, but this claim is not supported by a review of previous studies. The research gap is therefore stated rather than proven.

 Authors should clearly state:

- what is already known from previous research;
- what remains unexplored;
- why the current research is needed;
- how the study goes beyond the cited EUSPA 2023 survey;
- what the precise research objectives and research questions are.

3. The classification of enterprises is conceptually unclear

 The manuscript divides respondents into startups, scaleups, small enterprises and medium-sized enterprises. However, these categories are not necessarily mutually exclusive. A startup or scaleup can also be a small or medium-sized enterprise.

 Authors should explain:

- how each category was defined;
- how respondents were assigned to only one category;
- whether the classification was based on the number of employees, the age of the enterprise, the growth rate or self-classification;
- why micro-enterprises were not listed separately;
- whether the EU definition of SME was used;
- whether financial criteria were taken into account in addition to the number of employees.

The term “employment type” should not be used for these categories, as they describe the size or stage of development of the enterprise, not the type of employment of the respondent.

 4. There is an inconsistency in the classification of industrial ecosystems

 The methodology states that all fourteen EU industrial ecosystems are represented. However, retail seems to be missing from the list, although it is subsequently mentioned in the results section. At the same time, the category “Proximity, Social Economy & Civil Security” is presented as one merged category.

 Authors should provide a complete table containing:

 all ecosystem categories;

- the exact number and proportion of respondents in each category;
- the definition or official source of the classification;
- the categories that have been merged or renamed;
- the number of respondents by ecosystem and type of business.

This inconsistency must be corrected throughout the manuscript.

 5. Statistical analysis is too limited

 The study presents descriptive percentage results using pie charts but does not report inferential statistical tests. Yet the manuscript repeatedly claims that there are differences between types of businesses and industrial ecosystems.

 Descriptive differences alone do not prove that groups differ systematically. Authors should therefore either:

- perform appropriate inferential analyses; or
- unambiguously present the manuscript as an exploratory descriptive study and avoid wording suggesting statistically confirmed differences.

Depending on the questionnaire structure, chi-square tests, Fisher’s exact test for small numbers, effect measures such as Cramer’s V, ordinal regression, or multinomial logistic regression might be appropriate. Confidence intervals should also be reported for the main percentage results.

 Authors should report:

- statistical software used;
- analysis procedure;
- statistical significance level;
- effect sizes;
- how small or empty cells are treated;
- how multiple testing is accounted for;
- justification for any merging of response categories.

Comparisons across sectors are particularly problematic because some ecosystems contain very few respondents. For example, the aerospace and defense sector includes only eight respondents, as the authors themselves acknowledge. Percentage results based on such small groups can be unstable and should not be interpreted in the same way as results based on much larger groups.

 6. A separate Discussion section should be added

 The manuscript moves from descriptive results directly to a concise conclusion. The results sections already contain interpretive claims and recommendations, but these are not systematically linked to theory or previous research.

 A separate Discussion should:

- explain why awareness and adoption of the technologies remain low;
- interpret the dominant position of unclear ROI as a barrier;
- explain why medium-sized enterprises appear more prepared;
- discuss the difference between sustainability concerns and adoption of space technologies;
- consider why navigation and positioning are perceived as more relevant than Earth observation;
- compare the results with previous studies;
- distinguish theoretical, managerial and policy implications;
- discuss alternative explanations;
- avoid causal claims based on cross-sectional data.

**Minor comments**

 The manuscript should consistently use the term “space-related technologies” or “space data and services”, or clearly explain the relationship between these terms.

 For readers outside the space technology field, definitions of Earth observation, GNSS and satellite communications should be provided.

 Authors should distinguish between awareness, interest, intention, readiness and actual adoption of a technology. These terms are sometimes used interchangeably today.

 The figures contain a large number of small pie charts, making comparisons difficult. Differences would be better presented using tables or clustered bar graphs. Percentages should also include the exact number of respondents.

 All figures should include the number of observations, clear legends and sufficiently large captions. Authors should indicate whether percentages were calculated from the entire sample or only from valid responses to the relevant question.

 Figure 7 should clarify whether respondents selected one “biggest barrier” or whether they could indicate multiple barriers. The text lists five possible barriers, but the format of the response is not sufficiently explained.

 The phrase “surprisingly, half of respondents do not perceive a need” should be replaced with a neutral academic statement.

 The manuscript requires thorough language proofreading. Several sentences contain punctuation or grammatical errors.

 The limitations section should discuss bias caused by the common data collection method, respondents’ self-reports, social desirability, uneven sector representation, and the inability to establish causal relationships based on a cross-sectional design.

Is the study design appropriate and does the work have academic merit?

Partly

Is the work clearly and accurately presented and does it cite the current literature?

Partly

If applicable, is the statistical analysis and its interpretation appropriate?

No

Are all the source data underlying the results available to ensure full reproducibility?

Yes

Are the conclusions drawn adequately supported by the results?

Partly

Are sufficient details of methods and analysis provided to allow replication by others?

No

Reviewer Expertise:

My areas of expertise include business economics and management, small and medium-sized enterprises, sustainability and ESG, the green and digital transition, innovation and technology adoption, and the cultural and creative industries. I have experience with quantitative research methods, including correlation and regression analysis, panel-data analysis, factor analysis, and the evaluation of secondary statistical data.

I confirm that I have read this submission and believe that I have an appropriate level of expertise to confirm that it is of an acceptable scientific standard, however I have significant reservations, as outlined above.
